# Investigating Quantum Materials with Half-Polarized Diffraction and magnetic PDF analysis at the HB-2A Neutron Powder Diffractometer

**DOI:** 10.1063/4.0000894

**Published:** 2025-10-27

**Authors:** Stuart Calder, Raju Baral, Matthew Powell, Joseph Kolis, Haidong Zhou

**Affiliations:** 1 Oak Ridge National Laboratory, Oak Ridge, TN, USA; 2 Clemson University, Clemson, SC, USA; 3 University of Tennessee, Knoxville, TN, USA

## Abstract

Local magnetic ordering and anisotropy is often central to the emergent behavior and subsequent functional properties in quantum materials and beyond. Neutron powder diffraction provides a straightforward yet extremely powerful technique for quantitative measurements of microscopic magnetic properties. The HB-2A powder diffractometer located at the High Flux Isotope Reactor in ORNL is traditionally utilized for long-range magnetic structure determination. Recently these capabilities have been extended to include methods aimed at accessing local magnetism: Half- polarized neutron powder diffraction (pNPD) and magnetic pair distribution function (mPDF) analysis. These two distinct techniques are possible on HB-2A due to the versatility of the instrument’s reciprocal space coverage, resolution and novel ultra-low temperature multi-sample changers that operate down to dilution refrigerator temperatures. This provides unique capabilities not found on any powder diffraction instrument and is particularly well suited to investigations of magnetic quantum materials. The development and implementation of these techniques will be discussed with a series of science case examples ranging from geometric frustrated magnets to magnetic metal-organic frameworks. Data reduction and analysis tools will be presented that enable the extraction of the local site susceptibility tensor and local spin-spin correlations in real space. Finally, potential combinations of these techniques in the form of half-polarized magnetic pair distribution function (pmPDF) analysis will be considered. Looking forward, HB-2A is undergoing a detector upgrade that will be in the user program by 2026. This will offer an order of magnitude increase in count rates to further aid the development of these often low signal measurements and provide new scientific capabilities.

